# Schisandra chinensis inhibits the entry of BoHV-1 by blocking PI3K-Akt pathway and enhances the m6A methylation of gD to inhibit the entry of progeny virus

**DOI:** 10.3389/fmicb.2024.1444414

**Published:** 2024-07-22

**Authors:** Yang Liu, Kang Wang, Xiao Gong, Weijie Qu, Yangyang Xiao, Hongtao Sun, Jingli Kang, Jinliang Sheng, Faxing Wu, Feiyan Dai

**Affiliations:** ^1^College of Veterinary Medicine, Yunnan Agricultural University, Kunming, China; ^2^Key Laboratory of Animal Biosafety Risk Prevention and Control of Ministry of Agriculture and Rural Affairs (South), China Animal Health and Epidemiology Center, Qingdao, China; ^3^Qingdao YeBio Bio-Engineering Co., Ltd., Qingdao, China; ^4^College of Animal Science and Technology, Shihezi University, Xinjiang, China

**Keywords:** Schisandra chinensis, bovine herpesvirus-1, Methylgomisin O, endocytosis, methylation

## Abstract

Schisandra chinensis, a traditional Chinese medicine known for its antitussive and sedative effects, has shown promise in preventing various viral infections. Bovine herpesvirus-1 (BoHV-1) is an enveloped DNA virus that causes respiratory disease in cattle, leading to significant economic losses in the industry. Because the lack of previous reports on Schisandra chinensis resisting BoHV-1 infection, this study aimed to investigate the specific mechanisms involved. Results from TCID_50_, qPCR, IFA, and western blot analyses demonstrated that Schisandra chinensis could inhibit BoHV-1 entry into MDBK cells, primarily through its extract Methylgomisin O (Meth O). The specific mechanism involved Meth O blocking BoHV-1 entry into cells via clathrin- and caveolin-mediated endocytosis by suppressing the activation of PI3K-Akt signaling pathway. Additionally, findings from TCID_50_, qPCR, co-immunoprecipitation and western blot assays revealed that Schisandra chinensis blocked BoHV-1 gD transcription through enhancing m6A methylation of gD after virus entry, thereby hindering gD protein expression and preventing progeny virus entry into cells and ultimately inhibiting BoHV-1 replication. Overall, these results suggest that Schisandra chinensis can resist BoHV-1 infection by targeting the PI3K-Akt signaling pathway and inhibiting gD transcription.

## 1 Introduction

Schisandra chinensis (SC), a member of the Magnoliaceae Schisandra, is a plant species with a global distribution, primarily sourced from China (Hsu et al., 2012). It also referred to as “Wuweizi” or “Beiwuweizi” in China (Szopa et al., 2017), the dried ripe fruit of Schisandra chinensis has been utilized in traditional Chinese medicine for over 2000 years. It has been officially recognized in various editions of the Chinese pharmacopoeia and is commonly utilized in traditional Chinese medicine formulations (Li Z. et al., 2018; Szopa et al., 2018; Peng et al., 2022). Known for its antitussive and sedative properties (Zhong et al., 2016, 2021). Previous studies have also indicated that Schisandra polysaccharide (SPJ) exhibits inhibitory effects on influenza A virus both *in vitro* and *in vivo* (Qi et al., 2023), schinlignan G and methylgomisin O have shown potential in combating hepatitis B virus (Xue et al., 2015).

BoHV-1, a member of the α-herpesvirus subfamily, is an enveloped double-stranded DNA virus which infects various cattle, leading to severe respiratory and reproductive tract diseases (Edwards et al., 1990; van Oirschot, 1995; D'Arce et al., 2002). First reported in Switzerland in 1841, this virus is now globally distributed, causing significant economic losses in the cattle industry (Turin et al., 1999; Jones and Chowdhury, 2007). Many α-herpesvirus entry into host cells primarily occurs through endocytosis, such as Herpes simplex virus 1 (HSV-1), Bovine herpesvirus-1 (BoHV-1) and Feline Herpesvirus type 1 (FeHV-1; Hasebe et al., 2009; Liu and Cohen, 2015; Lv et al., 2018; Praena et al., 2020; Eisa et al., 2021; Ferrara et al., 2023; Liu et al., 2023). Previous studies have shown that during BoHV-1 entry, BoHV-1 gD protein binds to cell receptor, activating PI3K-Akt and p38 MAPK signaling pathways. This activation promotes the expression of clathrin, caveolin and dynamin, enhancing both clathrin-mediated and caveolin-mediated endocytosis, facilitating efficient virus entry into cells (Zhu et al., 2011, 2016; Liu et al., 2023).

Methylation is an epigenetic modification that plays a key role in the regulation of gene expression, genomic imprinting, embryonic development and the occurrence and development of tumors and diseases (Dai et al., 2021). N6-methyladenine (m6A) methylation is a common modification in eukaryotic mRNAs, affecting mRNA stability, translation, and export (Wang et al., 2014). During viral infection, the m6A modification levels of viral and host RNA will change, which affect the replication of virus and the state of cells. In addition, m6A modification plays different roles in different types of viral infections (An and Duan, 2022; Yue et al., 2022). Studies on the regulatory mechanism of m6A modification in the process of viral replication show that m6A modification regulates viral replication (Sun et al., 2019; Yue et al., 2022). It is achieved by affecting the stability of viral mRNA or genomic RNA, and the effect of m6A modification on the host is related to the immune system of the host in a certain degree (An and Duan, 2022).

Based on these reports, our study investigated the role of Schisandra chinensis in BoHV-1 infection of host cells.

## 2 Materials and methods

### 2.1 Cells and viruses

MDBK cells and MDBK-gD cells were provided by the China Animal Health and Epidemiology Center and cultured in DMEM medium (Thermo Fisher Scientific, Waltham, America) containing 10% FBS (ExCell Bio, Taicang, China). The BoHV-1 Shaanxi strain, which was isolated and identified in 2021 and provided by the China Animal Health and Epidemiology Center, was amplified in MDBK cells. The BoHV-1-EGFP virus, using homologous recombination to insert the EGFP gene between the gI and gD genes of the BoHV-1, was provided by the China Animal Health and Epidemiology Center and amplified in MDBK cells.

### 2.2 Antibodies and reagents

The rabbit anti-caveolin-1, anti-clathrin heavy chain (CHC), anti-PI3K p85 (phos pho Y458) + PI3 Kinase p55 (phospho Y199), anti-cGAS, anti-STING, anti-TBKl, anti-IRF3, anti-MAVS, anti-MDA5, anti-RIG-I, anti-N6-methyladenosine (m6A) monoclonal antibodies were purchased from Abcam (Abcam, Cambridge, UK). The mouse anti-glyceraldehyde3-phosphate dehydrogenase (GAPDH) monoclonal antibody was purchased from Abcam (Abcam, Cambridge, UK). The rabbit anti-dynamin-2 and anti-nectin-1 polyclonal antibody, goat anti-rabbit HRP-labeled secondary antibody, goat anti-mouse HRP-labeled secondary antibody was purchased from Abcam (Abcam, Cambridge, UK). The rabbit anti-phospho-Akt (Ser473), anti-Phospho-p38 MAPK (Thr180/Tyr182), anti-phospho-caveolin-1 (Tyr14) monoclonal antibodies were purchased from Cell Signaling Technology (Cell Signaling Technology, Danvers, USA). The mouse anti-BoHV-1 gD monoclonal antibody was provided by China Animal Health and Epidemiology Center. Bovine IFN-β ELISA kit was purchased from Shanghai Keshun science and technology Company Limited (Shanghai Keshun, Shanghai, China). NH_4_Cl was purchased from Sigma (Sigma, MO, USA). Chloroquine was purchased from MedChemExpress (MedChemExpress, Shanghai, China). Schisandrae chinensis was collected from Qingyuan County, Liaoning province, China. Methylgomisin O (Meth O), Angeloylgomisin H (Ange H), Rubrisandrins A (Rubr A), Schisanchinin B (Schi B) and Wuweilignan E (Wuwe E) were purchased from Huayuan Shopping Mall. CCK-8 reagent was purchased from Vazyme Biotech (Vazyme Biotech, Nanjing, China).

### 2.3 Preparation of Schisandrae chinensis

The Schisandrae chinensis were crushed, and 100 g of the Schisandrae chinensis powder was soaked in 2,000 ml of warm pure water for 2 h and boiled for 1 h. After cooling, the liquid was first filtered through medical gauze, and then passed through a 0.45 μm microporous filter membrane to obtain a Schisandra chinensis storage solution with a concentration of 50 mg/ml. This solution was further diluted with PBS to create different concentrations of working solution for subsequent experiments.

### 2.4 Chemical inhibitors and cell viability determination

MDBK cells were seeded into 96-well plates with DMEM containing 2% FBS. Once the cells reached full confluency, they were treated with Schisandrae chinensis or extracts for 24 h. Subsequently, CCK-8 reagent was introduced to the cells. Following a 4 h incubation at 37°C, the absorbance was measured at 450 nm using a microplate reader from Flash Spectrum Biotechnology in Shanghai, China.

### 2.5 Medicines treatment and viral replication

MDBK cells were seeded into 6-well plates and incubated for 24 h. Afterward, the cells were exposed to Schisandrae chinensis or extracts at specified concentrations for 1 h at 37°C, followed by inoculation with BoHV-1 at a multiplicity of infection (MOI) of 2 and incubated at 4°C for 1 h. Subsequently, the cells were washed with PBS to remove unabsorbed virus and maintained in DMEM with 2% FBS and inhibitor at 37°C. DMSO-treated MDBK cells served as the control. After 24 h, supernatants were collected for virus titration analysis, and the infected cells were treated with proteinase K at 4°C to eliminate non-internalized virus, followed by treatment with 2 mM phenylmethylsulfonyl fluoride (PMSF). Total DNA was then extracted from the cells and quantitated using qPCR analysis.

### 2.6 Virus attachment

MDBK cells were pretreated by Schisandrae chinensis or extracts for 1 h, the cells were inoculated with BoHV-1 at a multiplicity of infection (MOI) of 10 in the presence of medicine and were placed at 4°C for 1 h. The cells were washed repeatedly with PBS to remove the unabsorbed virus, The attached virus was collected by repeated freezing and thawing at −80°C for virus titer and virus copies analysis.

### 2.7 Virus entry

MDBK cells were pretreated by Schisandrae chinensis or extracts for 1 h, the cells were inoculated with BoHV-1 at an MOI of 10 and incubated at 4°C for 1 h. After washing with PBS to remove unabsorbed virus, the cells were cultured in DMEM with 2% FBS and medicine at 37°C for 1 h. Subsequently, the cells were harvested, treated with proteinase K at 4°C to eliminate non-internalized virus, and then exposed to 2 mM phenylmethylsulfonyl fluoride (PMSF). Total DNA was extracted from the cells and quantified using qPCR analysis.

### 2.8 TCID_50_ analysis

MDBK cells were seeded in 96-well plates and allowed to reach confluence. A serial 10-fold dilution of BoHV-1 solution was then prepared and added to the plates, followed by incubation at 37°C for 1 h. Subsequently, the cells were washed with PBS, replenished with 100 μl of 2% FBS DMEM, and incubated at 37°C for 5 to 7 days to count the total number of CPE holes. The TCID_50_ was determined using the Reed-Muench formula (Thakur and Fezio, 1981).

### 2.9 qPCR

Cells were harvested, RNA was extracted, followed by reverse transcription. The real-time PCR system (TIANLONG, Xian, China) was used to detect BoHV-1 copies and measure the mRNA relative expression of CHC, dynamin-2, caveolin-1, IFN-α, IFN-β, and BoHV-1 gD. The primer and probe sequences utilized for qPCR can be found in Table 1.

**Table 1:** The sequences of the primers and probe.

siRNA name	Sequence (5′-3′)
BoHV-1-gE qF	TAACAGCGTAGACCTGGTCT
BoHV-1-gE qR	TCCGAAGTAACGACTAGGCT
BoHV-1-gE Probe	CGTCTTTGTGCTGCAGTACAAC
CHC-qF	AATGTTATGCGTATCAGTCC
CHC-qR	AGATGGGCGATTATTCTTCA
Dynamin-qF	AAGCACGTCTTCGCCATCTT
Dynamin-qR	CTGGGCTCCATCCTCATTTT
Caveolin-1-qF	AGGAAATGAACGAGAAGCAA
Caveolin-1-qR	CACAGTGAAGGTGGTGAAGC
IFN-α-qF	GTCCTGATGCTCCTGAGACAA
IFN-α-qR	GTGCTGAAGAGCTGGAAGGT
IFN-β-qF	CCTGTGCCTGATTTCATCATGA
IFN-β-qR	GCAAGCTGTAGCTCCTGGAAAG
BoHV-1-gD-qF	ACCGAGTGCGAGCCCAGGAA
BoHV-1-gD-qR	GCGACCGTGCCGTCGATGTA
MeRIP-BoHV-1-gD-qF	CCCGTTTTGGGACAGCTTC
MeRIP-BoHV-1-gD-qR	CGCCGAGTTTCGAGAACCA
GAPDH-qF	TCGTCGCCATCAATGACCCCT
GAPDH-qR	CTCAGCACCAGCATCACCCCA

### 2.10 Western blot and gray scale analysis

Protein samples ran on 10% SDS-PAGE gels and were subsequently transferred to polyvinylidene fluoride (PVDF) membranes. The PVDF membranes were then incubated with 5% milk for 1 h at room temperature, followed by primary antibody incubation overnight at 4°C, and secondary antibody incubation for 1 h at room temperature. Protein bands were analyzed using ECL chemiluminescence reagent (Monad, Suzhou, China) and a chemiluminescence analyzer (UVITEC, Cambridge, England). The target bands were subjected to gray scale analysis by Image J 1.54d.

### 2.11 Co-immunoprecipitation

400 μl of cell lysates were incubated with 1 μg of primary antibody overnight at 4°C. Subsequently, 20 μl of Protein A + G Agarose was added and the mixture was gently shaken for 3 h at 4°C. The supernatant was removed after centrifugation, and the precipitate was washed multiple times with PBS. Following that, SDS-PAGE electrophoresis buffer was added and the sample was heated at 95°C for 10 min for immunoblot analysis.

### 2.12 Methylated RNA immunoprecipitation coupled with qPCR

Cells were harvested and total RNA was extracted using RNAiso Plus (Takara, Beijing, China). Subsequently, RNA was fragmented utilizing the m6A MeRIP kit (Cloud Sequence Biotechnology, Shanghai, China), with 3 μg of fragmented RNA serving as the input sample. Following immunoprecipitation with m6A antibody, the RNA was extracted and the methylation level of m6A was assessed using qPCR.

### 2.13 Progeny virus replication and entry

MDBK cells were pretreated by Schisandrae chinensis for 1 h, the cells were inoculated with BoHV-1 at an MOI of 10 and incubated at 4°C for 1 h. After washing with PBS to remove unabsorbed virus, the cells were cultured in DMEM with 2% FBS and medicine at 37°C. 24 h later, the supernatant was collected and reinoculated into MDBK cells. The cells were infected at 4°C for 1 h, transfer to 37°C for 24 h to detect virus replication and transfer to 37°C for 1 h to detect virus entry.

### 2.14 Statistical analyses

The experimental results were obtained from three independent experiments and analyzed using one-way ANOVA with Graphpad Prism Version 5.0 software. Asterisks indicate the significance of differences, with *p < 0.05, **p < 0.01, and ***p < 0.001.

## 3 Results

### 3.1 Schisandra chinensis inhibits BoHV-1 replication

**Figure 1:**
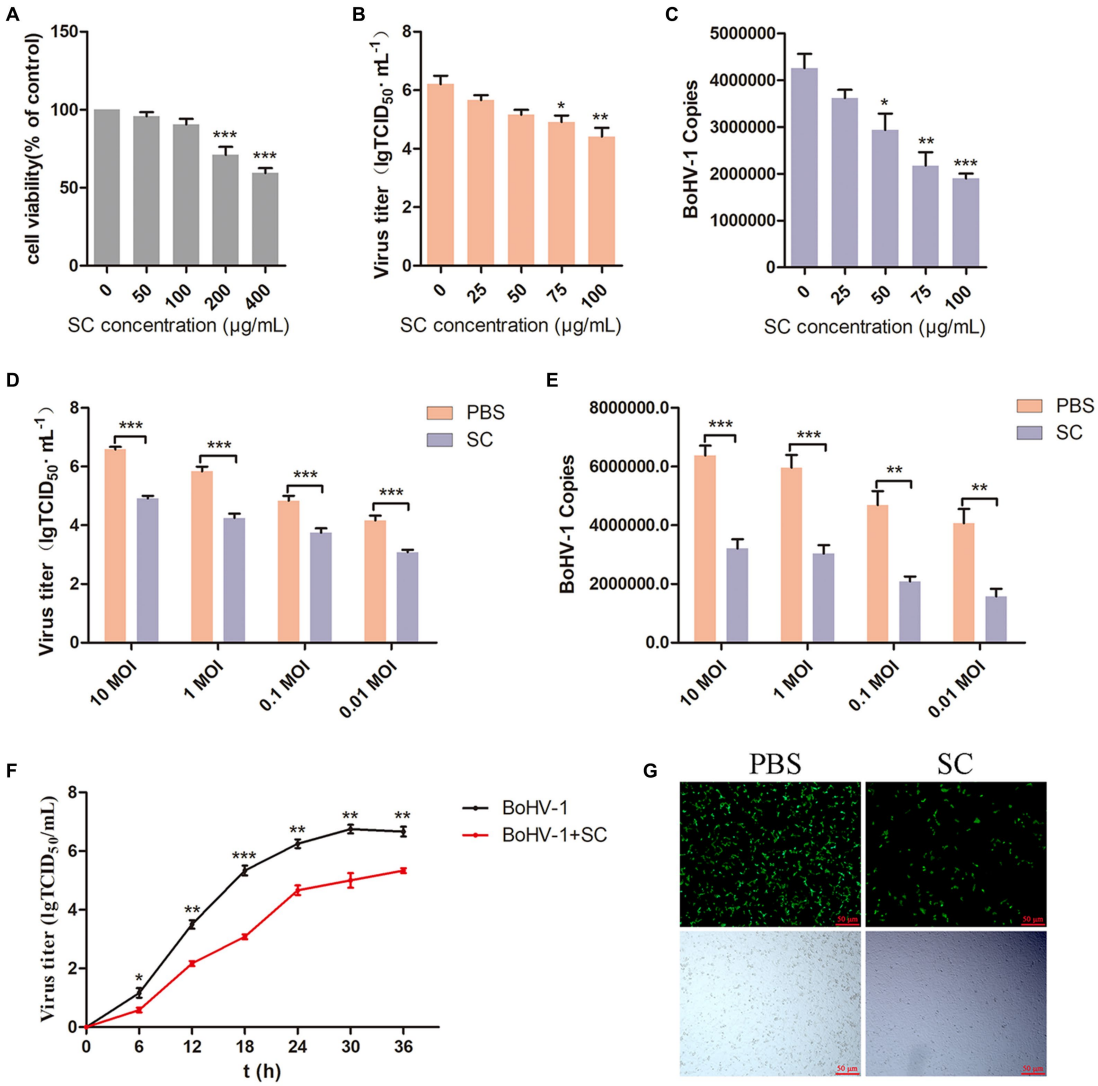
Schisandra chinensis is involved in BoHV-1 replication. **(A)** Viability of MDBK cells treated with Schisandra chinensis (SC) were analyzed by CCK-8 reagent. **(B)** MDBK cells were pretreated with various concentrations of SC for 1 h at 37°C, followed by bound with BoHV-1 at an MOI of 2 for 1 h at 4°C. Subsequently, the cells were transferred to 37°C for 24 h in the presence of SC. Supernatant was collected to analyze virus replication by virus titer, **(C)** cells were collected to analyze virus replication by virus copies. **(D)** MDBK cells were pretreated with 100 μg/ml SC for 1 h at 37°C, followed by bound with different concentrations of BoHV-1 for 1 h at 4°C, and then transferred to 37°C for 24 h in the presence of SC. Supernatants were collected to analyze BoHV-1 replication by viral titer, **(E)** cells were collected to analyze BoHV-1 replication by viral copies. **(F)** MDBK cells were pretreated with SC at 37°C for 1 h and inoculated with 2 MOI BoHV-1 for 1 h. After 1 h, the supernatant was discarded and replaced with DMEM containing 2% FBS. Supernatants from different time periods were collected and virus titer was measured. **(G)** MDBK cells were pretreated with 100 μg/ml SC at 37°C for 1 h, then bound with BoHV-1-EGFP at an MOI of 2 at 4°C for 1 h, followed by transfer to 37°C for incubation in the presence of SC. 24 h later, the number of infected cells was observed under a fluorescence microscope. *p < 0.05, **p < 0.01, and ***p < 0.001.

To evaluate the antiviral effect of Schisandra chinensis on BoHV-1, we initially determined the appropriate adding concentration of Schisandra chinensis. Cell viability assessment indicated that Schisandra chinensis greater than 100 μg/ml reduce the viability of MDBK cells (Figure 1A). MDBK cells were pretreated with different concentrations of Schisandra chinensis at 37°C for 1 h, followed by infection with BoHV-1 at 4°C for 1 h to allow virus attachment in the presence of Schisandra chinensis. After removing unbound virus, infected cells were cultured at 37°C for 24 h to assess virus replication. The results of virus titer showed that Schisandra chinensis inhibited BoHV-1 replication in a dose-dependent manner (Figure 1B), which was corroborated by virus copies (Figure 1C). Further investigation involved infecting MDBK cells with different BoHV-1 doses to ascertain if the inhibitory effect of Schisandra chinensis on replication was influenced by the virus dose. Notably, the inhibitory impact of Schisandra chinensis on BoHV-1 replication remained consistent across varying infection doses, as evidenced by virus titer and virus copies results (Figures 1D,E). Furthermore, we tested the virus growth curve of BoHV-1 after the addition of Schisandra chinensis, and the growth curve of BoHV-1 without the addition of Schisandra chinensis was used as a control. Results indicated that Schisandra chinensis led to a decrease in virus titer during early infection stage, and the virus titer remained notably lower than the control during subsequent replication (Figure 1F). To further investigate the role of Schisandra chinensis in BoHV-1 replication, MDBK cells were treated with Schisandra chinensis followed by infection with BoHV-1-EGFP for 24 h and detecting cell fluorescence. The results showed that the number of infected cells was significantly reduced after the addition of Schisandra chinensis (Figure 1G). These results suggest that Schisandra chinensis inhibits BoHV-1 replication.

### 3.2 Schisandra chinensis inhibits BoHV-1 entry into MDBK cells

**Figure 2:**
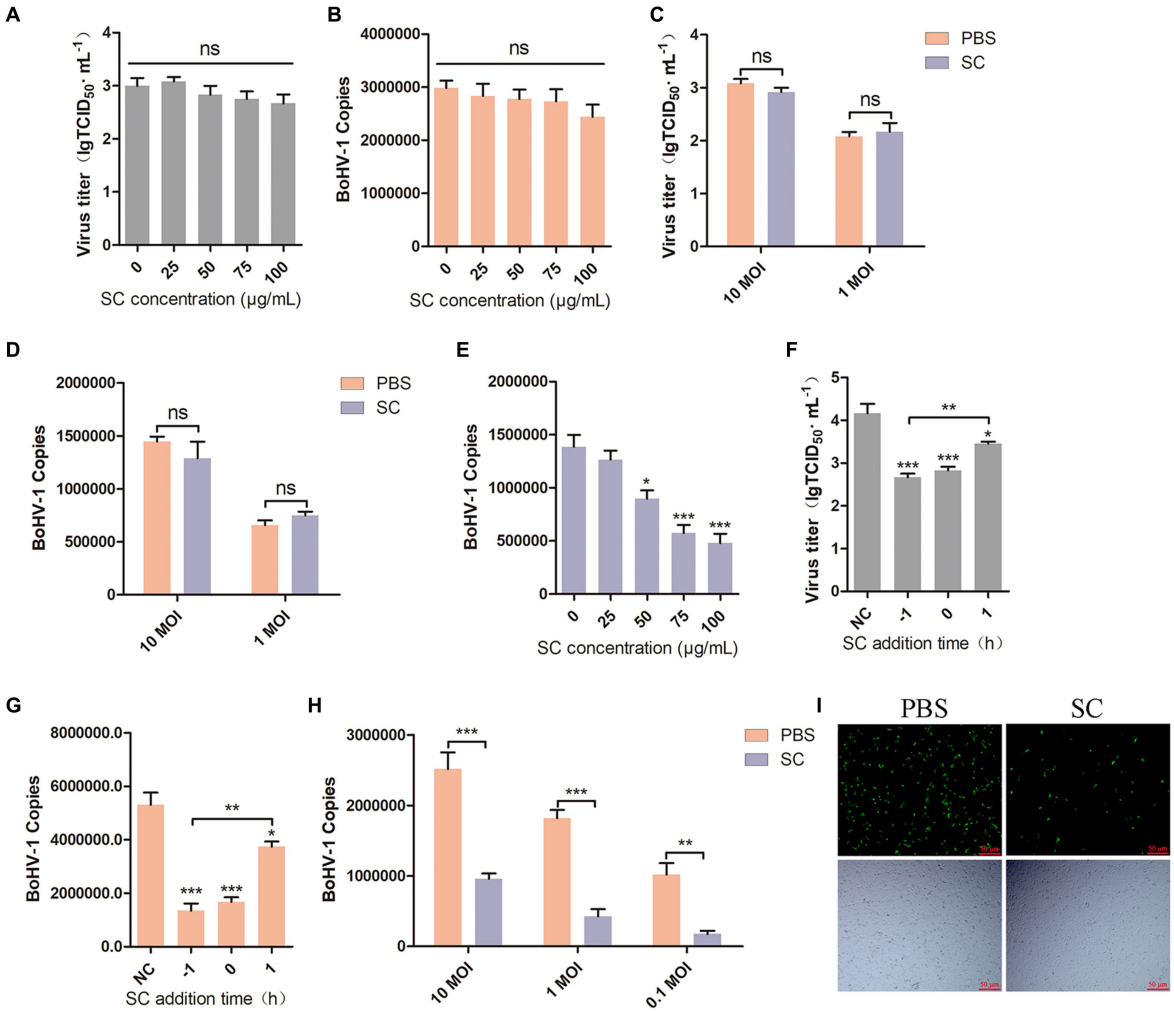
Schisandra chinensis inhibits BoHV-1 entry into MDBK cells. **(A)** MDBK cells were pretreated with various concentrations of SC for 1 h at 37°C, followed by bound with BoHV-1 at an MOI of 10 for 1 h at 4°C. The cells were repeatedly freeze-thawed at −80°C, the attachment was analyzed by virus titer, **(B)** the attachment was analyzed by virus copies. **(C)** MDBK cells were pretreated with 100 μg/ml SC for 1 h at 37°C, followed by bound with different concentrations of BoHV-1 for 1 h at 4°C. The cells were repeatedly freeze-thawed at −80°C, the attachment was analyzed by virus titer, **(D)** the attachment was analyzed by virus copies. **(E)** MDBK cells were pretreated with various concentrations of SC for 1 h at 37°C, followed by bound with BoHV-1 at an MOI of 10 for 1 h at 4°C. Subsequently, the cells were transferred to 37°C for 1 h in the presence of SC. Cells were collected to analyze virus entry by virus copies. **(F)** SC was applied either 1 h before or after BoHV-1 infection. MDBK cells were exposed to BoHV-1 at 4°C for 1 h and then shifted to 37°C for 24 h. Virus entry and replication were assessed through virus titer, **(G)** Virus entry and replication were assessed through virus copies. **(H)** MDBK cells were pretreated with 100 μg/ml SC for 1 h at 37°C, followed by bound with different concentrations of BoHV-1 for 1 h at 4°C. Subsequently, the cells were transferred to 37°C for 1 h in the presence of SC. The cells were collected to analyze virus replication by virus copies. **(I)** MDBK cells were pretreated with 100 μg/ml SC at 37°C for 1 h, then bound with BoHV-1-EGFP at an MOI of 10 at 4°C for 1 h, followed by transfer to 37°C for incubation in the presence of SC. 1 h later, the number of infected cells was observed under a fluorescence microscope. *p < 0.05, **p < 0.01, and ***p < 0.001.

Viral attachment and entry are crucial processes in the viral life cycle (Choppin and Scheid, 1980; Mercier et al., 2008), BoHV-1 enters the cell to facilitate its proliferation (Yamauchi and Helenius, 2013). To investigate the impact of Schisandra chinensis on BoHV-1 attachment, MDBK cells were pretreated with varying concentrations of Schisandra chinensis at 37°C for 1 h. Subsequently, the cells were infected with BoHV-1 at 4°C for 1 h to allow the virus to attach to the cell surface in the presence of Schisandra chinensis. The unbound virus was then removed, and the attachment of the virus was assessed. The results from virus titer and virus copies analysis indicated that Schisandra chinensis did not affect BoHV-1 attachment (Figures 2A,B). We examined the effect of Schisandra chinensis on BoHV-1 attachment at different viral infection doses, virus titer and virus copies showed that Schisandra chinensis had no effect on BoHV-1 attachment at different virus doses (Figures 2C,D). Next, we investigated the effect of Schisandra chinensis on BoHV-1 entry. MDBK cells were pre-treated with varying concentrations of Schisandra chinensis at 37°C for 1 h, followed by infection with BoHV-1 at 4°C for 1 h. After removing unbound virus, cells were further incubated at 37°C for 1 h to assess virus entry using qPCR. The results indicated a dose-dependent inhibition of BoHV-1 entry by Schisandra chinensis (Figure 2E). The role of Schisandra chinensis in both BoHV-1 entry and replication was explored by treating MDBK cells with Schisandra chinensis 1 h before and after BoHV-1 inoculation. The results of virus titer and virus copies showed that Schisandra chinensis inhibited BoHV-1 entry and replication process (Figures 2F,G). We examined the impact of varying BoHV-1 inoculation concentrations on the inhibitory effect of Schisandra chinensis on BoHV-1 entry. The result of virus copies revealed that Schisandra chinensis inhibited BoHV-1 entry into MDBK cells across different virus inoculation doses (Figure 2H). Furthermore, we further investigate the role of Schisandra chinensis in virus entry, MDBK cells were treated with Schisandra chinensis and cellular fluorescence was examined 1 h after infection with BoHV-1-EGFP virus. The findings indicated a significant reduction in the number of infected cells following Schisandra chinensis treatment (Figure 2I). These results imply that Schisandra chinensis inhibits BoHV-1 entry into MDBK cells.

### 3.3 Schisandra chinensis blocks BoHV-1 replication and entry by inhibiting endocytosis

**Figure 3:**
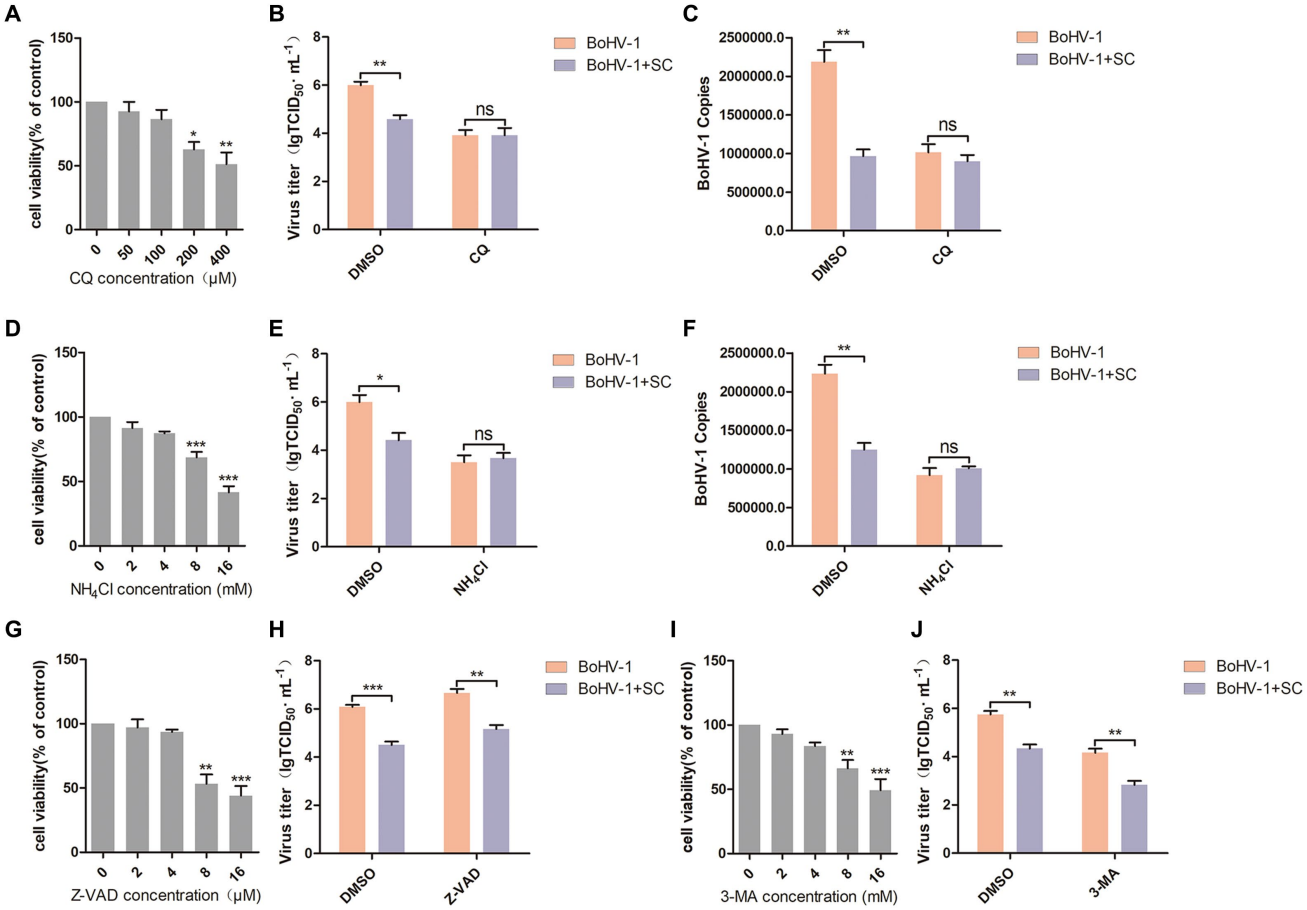
Schisandra chinensis inhibits endocytosis to block BoHV-1 replication and entry. **(A,D,G,I)** Viability of MDBK cells treated with CQ/NH_4_Cl/Z-VAD/3-MA were analyzed by CCK-8 reagent. **(B)** MDBK cells were pre-treated with SC in combination with either CQ or DMSO for 1 h, followed by assessment of viral replication at 24 h post-inoculation with BoHV-1 through virus titer, **(C)** assessment of viral entry at 1 h post-inoculation with BoHV-1 through virus copies. **(E)** MDBK cells were pre-treated with SC in combination with either NH_4_Cl or DMSO for 1 h, followed by assessment of viral replication at 24 h post-inoculation with BoHV-1 through virus titer, **(F)** assessment of viral entry at 1 h post-inoculation with BoHV-1 through virus copies. **(H)** MDBK cells were pre-treated with SC in combination with either Z-VAD or DMSO for 1 h, followed by assessment of viral replication at 24 h post-inoculation with BoHV-1 through virus titer. **(J)** MDBK cells were pre-treated with SC in combination with either 3-MA or DMSO for 1 h, followed by assessment of viral replication at 24 h post-inoculation with BoHV-1 through virus titer. *p < 0.05, **p < 0.01, and ***p < 0.001.

Most enveloped viruses enter host cells through endocytosis, relying on the acidic environment of endosomes (Más and Melero, 2013; Nicola, 2016), including BoHV-1 (Liu et al., 2023). In this study, two inhibitors of endosomal acidification, chloroquine (CQ) and NH_4_Cl, were evaluated their optimal adding concentrations. Cell viability assays revealed that CQ greater than 100 μM and NH_4_Cl greater than 4 mM reduced the viability of MDBK cells (Figures 3A,D). MDBK cells were pre-treated with Schisandra chinensis in combination with either CQ or DMSO, followed by assessment of viral replication and entry at 24 and 1 h post-inoculation with BoHV-1. The results of virus titer indicated that Schisandra chinensis could inhibit BoHV-1 replication, and addition of CQ eliminates the inhibitory impact of Schisandra chinensis on BoHV-1 replication (Figure 3B). Similarly, virus copies analysis demonstrated that Schisandra chinensis hindered BoHV-1 entry, which was eliminated in the presence of CQ (Figure 3C). To further demonstrate this result, MDBK cells were pretreated with Schisandra chinensis and NH_4_Cl or DMSO together before observing viral replication and entry after inoculation with BoHV-1. The viral titer assay results indicated that NH_4_Cl eliminated the inhibitory effect of Schisandra chinensis on BoHV-1 replication (Figure 3E), while the viral copies assay results showed that NH_4_Cl eliminated the inhibitory effect of Schisandra chinensis on BoHV-1 entry (Figure 3F), suggesting that Schisandra chinensis hinders BoHV-1 replication and entry by inhibiting endocytosis. In addition, we also explored the involvement of apoptosis and autophagy in the inhibition of BoHV-1 replication by Schisandra chinensis. We examined the appropriate adding concentrations of apoptosis inhibitor Z-VAD and autophagy inhibitor 3-MA, the results of cell viability assay showed that Z-VAD greater than 4 μM and 3-MA greater than 4 mM could inhibit the viability of MDBK cells (Figures 3G,I). We pretreated MDBK cells with Schisandra chinensis in combination with either Z-VAD or DMSO followed by observation of virus replication after BoHV-1 inoculation. Virus titer showed that Z-VAD did not impact the inhibitory effect of Schisandra chinensis on BoHV-1 replication (Figure 3H). Similarly, 3-MA significantly reduced BoHV-1 replication but did not alter the inhibitory effect of Schisandra chinensis (Figure 3J). These results indicate that apoptosis and autophagy are not involved in the inhibition of BoHV-1 replication by Schisandra chinensis.

### 3.4 Methylgomisin O inhibited BoHV-1 entry into MDBK cells

**Figure 4:**
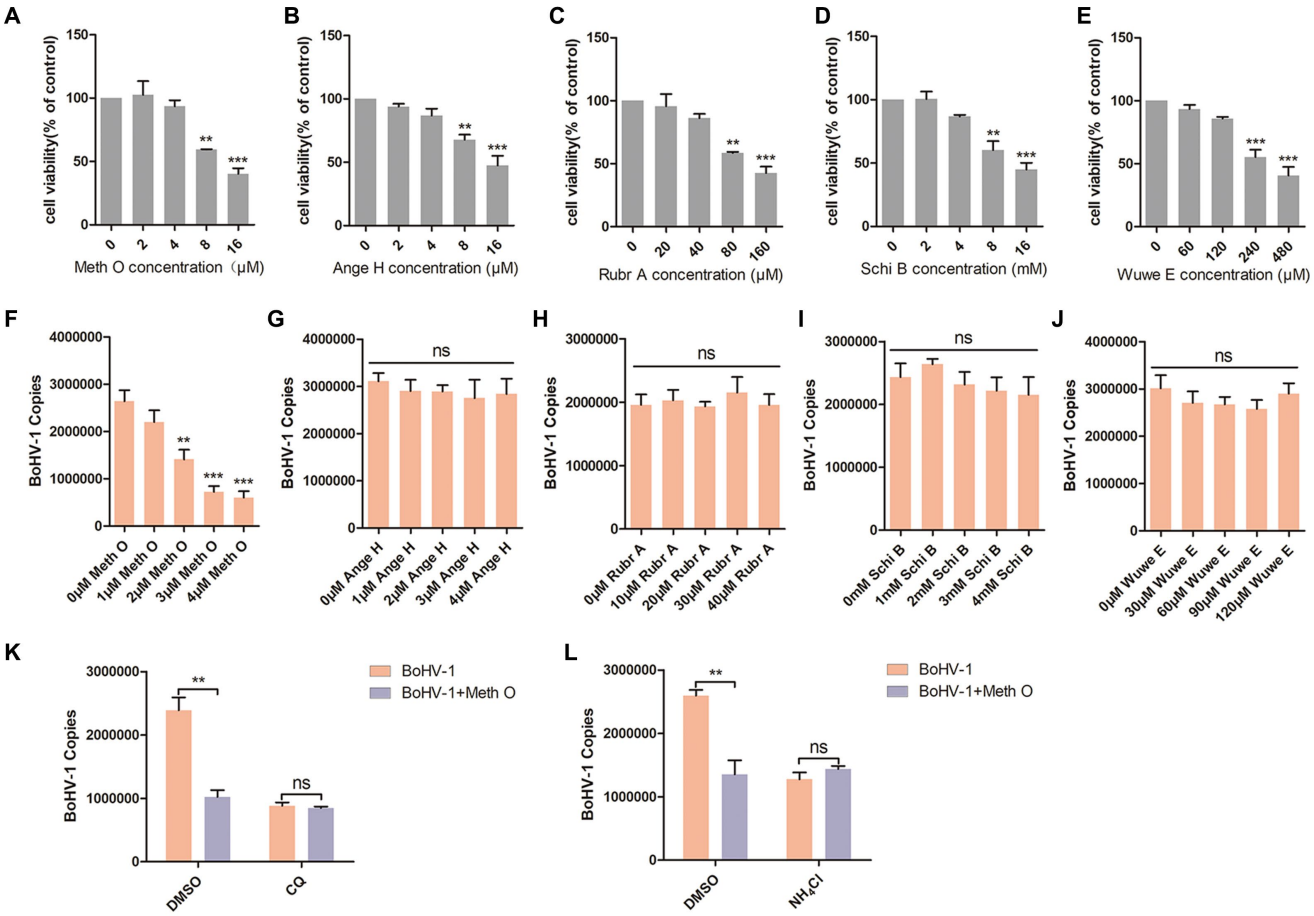
Methylgomisin O inhibited BoHV-1 entry into MDBK cells. **(A–E)** Viability of MDBK cells treated with Meth O, Ange H, Rubr A, Schi B or Wuwe E were analyzed by CCK-8 reagent. **(F–J)** MDBK cells were pretreated with various concentrations of Meth O/Ange H/Rubr A/Schi B/Wuwe E for 1 h at 37°C, followed by bound with BoHV-1 at an MOI of 10 for 1 h at 4°C. Subsequently, the cells were transferred to 37°C for 1 h. Cells were collected to analyze virus entry by virus copies. **(K)** MDBK cells were pre-treated with Meth O in combination with either CQ or DMSO for 1 h, followed by assessment of viral entry at 1 h post-inoculation with BoHV-1 through virus copies. **(L)** MDBK cells were pre-treated with Meth O in combination with either NH_4_Cl or DMSO for 1 h, followed by assessment of viral entry at 1 h post-inoculation with BoHV-1 through virus copies. **p < 0.01, and ***p < 0.001.

Previous reports have identified Methylgomisin O (Meth O), Angeloylgomisin H (Ange H), Rubrisandrins A (Rubr A), Schisanchinin B (Schi B) and Wuweilignan E (Wuwe E) as the extracts of Schisandra chinensis. In this study, we investigated the impact of these extracts on the entry of BoHV-1 into MDBK cells. The optimal working concentrations of Meth O, Ange H, Rubr A, Schi B, and Wuwe E were determined through cell viability assays, revealing that Meth O greater than 4 μM, Ange H greater than 4 μM, Rubr A greater than 40 μM, Schi B greater than 4 mM and Wuwe E greater than 120 μM inhibited the viability of MDBK cells (Figures 4A–E). Subsequent analysis of BoHV-1 entry using qPCR demonstrated that Meth O effectively inhibited the entry of BoHV-1 into MDBK cells (Figure 4F), Ange H, Rubr A, Schi B, and Wuwe E did not exhibit a significant impact on BoHV-1 entry (Figures 4G–J). To investigate whether Meth O inhibits BoHV-1 entry by blocking endocytosis, we pretreated MDBK cells with Meth O in combination with either chloroquine (CQ) or DMSO, and then observed virus entry 1 h after inoculation with BoHV-1. The result of virus copies detection showed that CQ could eliminate the inhibitory effect of Meth O on BoHV-1 entry (Figure 4K). Similarly, NH_4_Cl abolished the inhibitory effect of Meth O on BoHV-1 entry (Figure 4L). These results provide that Meth O blocks BoHV-1 entry by inhibiting endocytosis.

### 3.5 Methylgomisin O inhibits clathrin-mediated and caveolin-mediated endocytosis by blocking PI3K-Akt pathway

**Figure 5:**
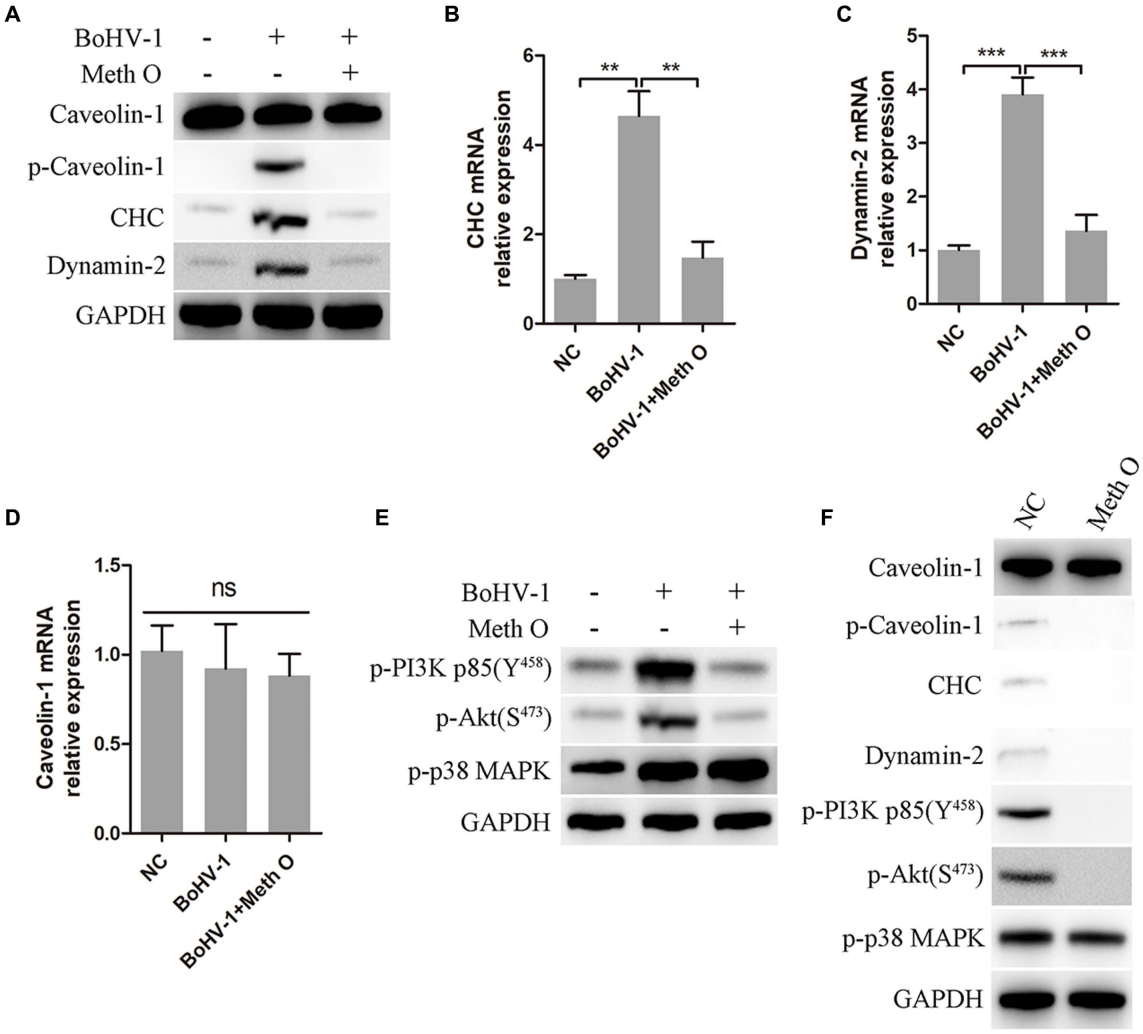
Methylgomisin O inhibits expression of clathrin, p-caveolin, dynamin-2, p-PI3K, p-Akt and p-p38 MAPK. **(A)** MDBK cells were pretreated with Meth O for 1 h at 37°C, followed by bound with BoHV-1 at an MOI of 10 for 30 min at 37°C. Subsequently, the cells were collected and detected by western blot with designated antibodies. **(B)** MDBK cells were pretreated with Meth O for 1 h at 37°C, followed by bound with BoHV-1 at an MOI of 10 for 30 min at 37°C. The expression of CHC mRNA was detected by qPCR. **(C)** The expression of dynamin-2 mRNA was detected by qPCR. **(D)** The expression of caveolin-1 mRNA was detected by qPCR. **(E)** MDBK cells were pretreated with Meth O for 1 h at 37°C, followed by bound with BoHV-1 at an MOI of 10 for 30 min at 37°C. Subsequently, the cells were collected and detected by western blot with designated antibodies. **(F)** MDBK cells were pretreated with Meth O for 90 min at 37°C, the cells were collected and detected by western blot with designated antibodies. **p < 0.01 and ***p < 0.001.

Previous research has indicated that BoHV-1 can enter MDBK cells through clathrin-mediated and caveolin-mediated endocytosis (Liu et al., 2023). In our study, we investigated the impact of Meth O on the expression of caveolin-1, clathrin heavy chain (CHC), and dynamin-2 in MDBK cells during early infection of BoHV-1. The results of western blot and gray scale analysis showed that BoHV-1 increased the expression of p-caveolin-1, CHC, and dynamin-2 at the initial infection stage (Liu et al., 2023), while not affecting the expression of caveolin-1 (Figure 5A), which was consistent with the report (Liu et al., 2023). Addition of Meth O significantly inhibited the expression of p-caveolin-1, CHC, and dynamin-2 (Figure 5A). Subsequent qPCR analysis revealed that Meth O suppressed the upregulation of CHC and dynamin-2 mRNA expression induced by BoHV-1 (Figures 5B,C), without affecting caveolin-1 mRNA expression (Figure 5D). BoHV-1 activate PI3K-Akt and p38 MAPK pathways to promote the expression of p-caveolin-1, CHC and dynamin-2, further promote clathrin-mediated and caveolin-mediated endocytosis to enhance BoHV-1 entry into MDBK cells (Zhu et al., 2011; Liu et al., 2023). We examined the effect of Meth O on PI3K-Akt and p38 MAPK pathways during BoHV-1 entry. Western blot analysis showed that the expression of p-PI3K, p-Akt and p-p38 MAPK was activated during BoHV-1 entry (Figure 5E). Upon Meth O treatment, the expression of p-PI3K and p-Akt was inhibited, but the expression of p-p38 MAPK remained unaffected (Figure 5E). These findings collectively suggest that Meth O inhibits p-caveolin-1, CHC and dynamin-2 expression by blocking the PI3K-Akt pathway, which further inhibits clathrin-mediated and caveolin-mediated endocytosis. To further support our conclusion, we explored the effect of Meth O on the expression of caveolin-1, p-caveolin-1, CHC, dynamin-2, p-PI3K, p-Akt and p-p38 MAPK without inoculating BoHV-1. The results indicate that Meth O can also suppress the above proteins expression in cells in the absence of virus infection (Figure 5F).

### 3.6 Schisandra chinensis does not affect IFN-I response

**Figure 6:**
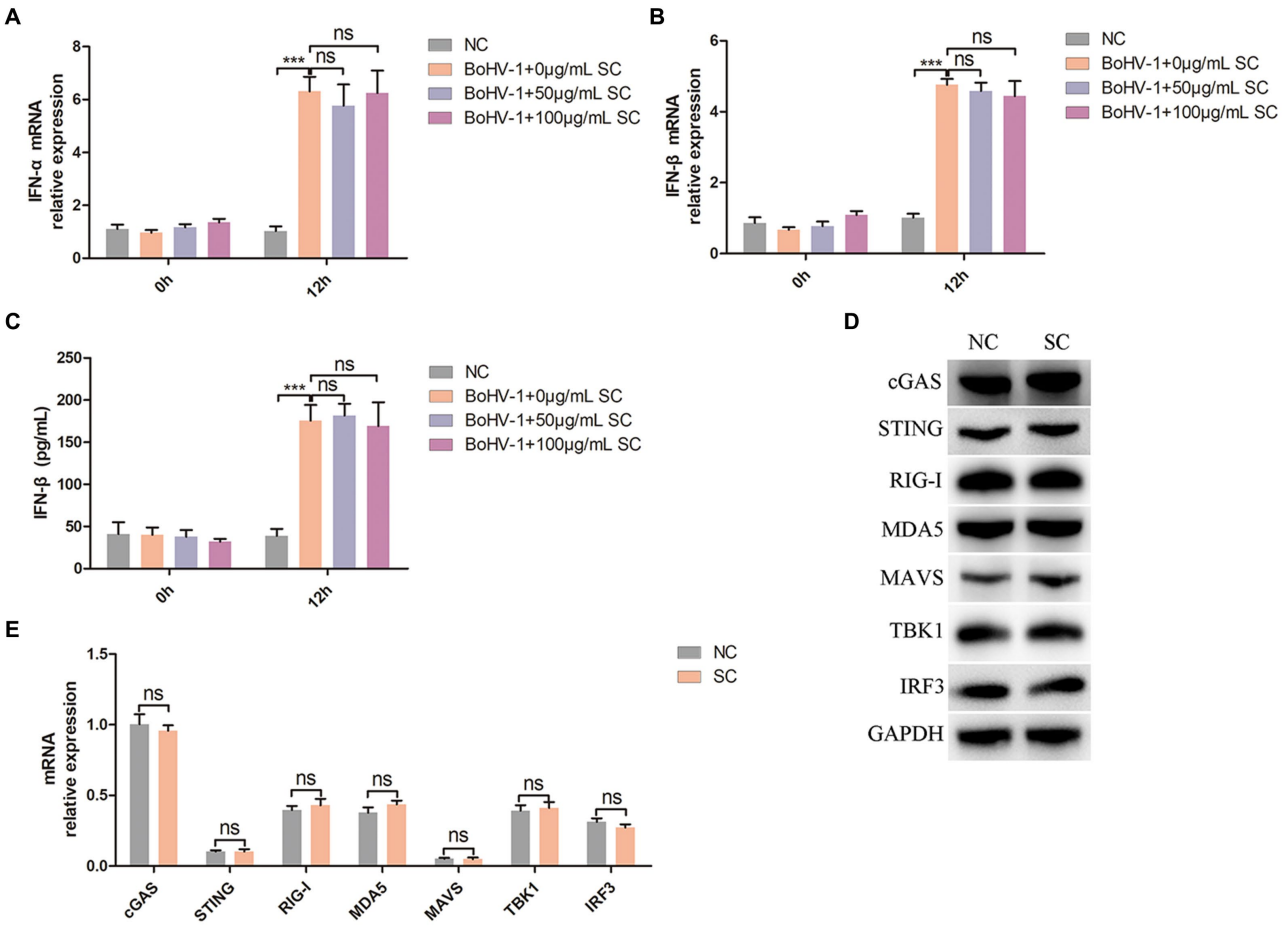
Schisandra chinensis does not affect IFN-I production. **(A)** MDBK cells were pretreated with SC for 1 h at 37°C, followed by bound with BoHV-1 at an MOI of 2 for 1 h or 12 h at 37°C. Subsequently, the cells were collected and detected the expression of IFN-α mRNA by qPCR, **(B)** detected the expression of IFN-β mRNA by qPCR, **(C)** and detected the production of IFN-β by ELISA. **(D)** MDBK cells were pretreated with PBS (NC) or SC for 12 h, the cells were collected and detected the protein expression by western blot. **(E)** MDBK cells were pretreated with PBS (NC) or SC for 12 h, the cells were collected and detected the expression of mRNA by qPCR. ***p < 0.001.

PI3K-Akt signaling regulate nuclear translocation of NF-κB and promote clathrin-mediated and caveolin-mediated endocytosis during BoHV-1 entry into MDBK cells (Liu et al., 2023). Mislocalized or unmasked host RNA serves as a potent RLR agonist during DNA virus infection (Chiang et al., 2018; Zhao et al., 2018; Lee et al., 2019; Choi et al., 2020), leading to NF-κB activation and subsequent IFN-I secretion (Iwasaki and Pillai, 2014; Thoresen et al., 2021). Therefore, we examined whether Schisandra chinensis inhibit IFN-I production by inhibiting nuclear translocation of NF-κB. Treatment of MDBK cells with different concentrations of Schisandra chinensis did not impact IFN-α and IFN-β production activated by BoHV-1 inoculation, as evidenced by qPCR and ELISA results (Figures 6A–C). Mislocalized or unmasked host RNA promoting IFN-I production not only activated by nuclear translocation of NF-κB, but also activated by RIG-I/MDA5-MAVS-TBK1-IRF3 signaling (Iwasaki and Pillai, 2014; Thoresen et al., 2021). In addition, upon recognition of viral DNA by host cells, cGAS-STING signaling was activated to promote IFN-I production (Chen et al., 2022; Erttmann et al., 2022). Western blot analysis revealed that Schisandra chinensis did not affect the protein expression levels of cGAS, STING, RIG-I, MDA5, MAVS, TBK1, and IRF3 (Figure 6D). This result was demonstrated by mRNA expression again (Figure 6E). Overall, while Schisandra chinensis inhibited NF-κB nuclear translocation, it did not affect RIG-I/MDA5-MAVS-TBK1-IRF3, and cGAS-STING-TBK1-IRF3 signaling, and therefore did not impact total IFN-I secretion.

### 3.7 Schisandra chinensis inhibits BoHV-1 gD transcription through enhancing its m6A methylation

**Figure 7:**
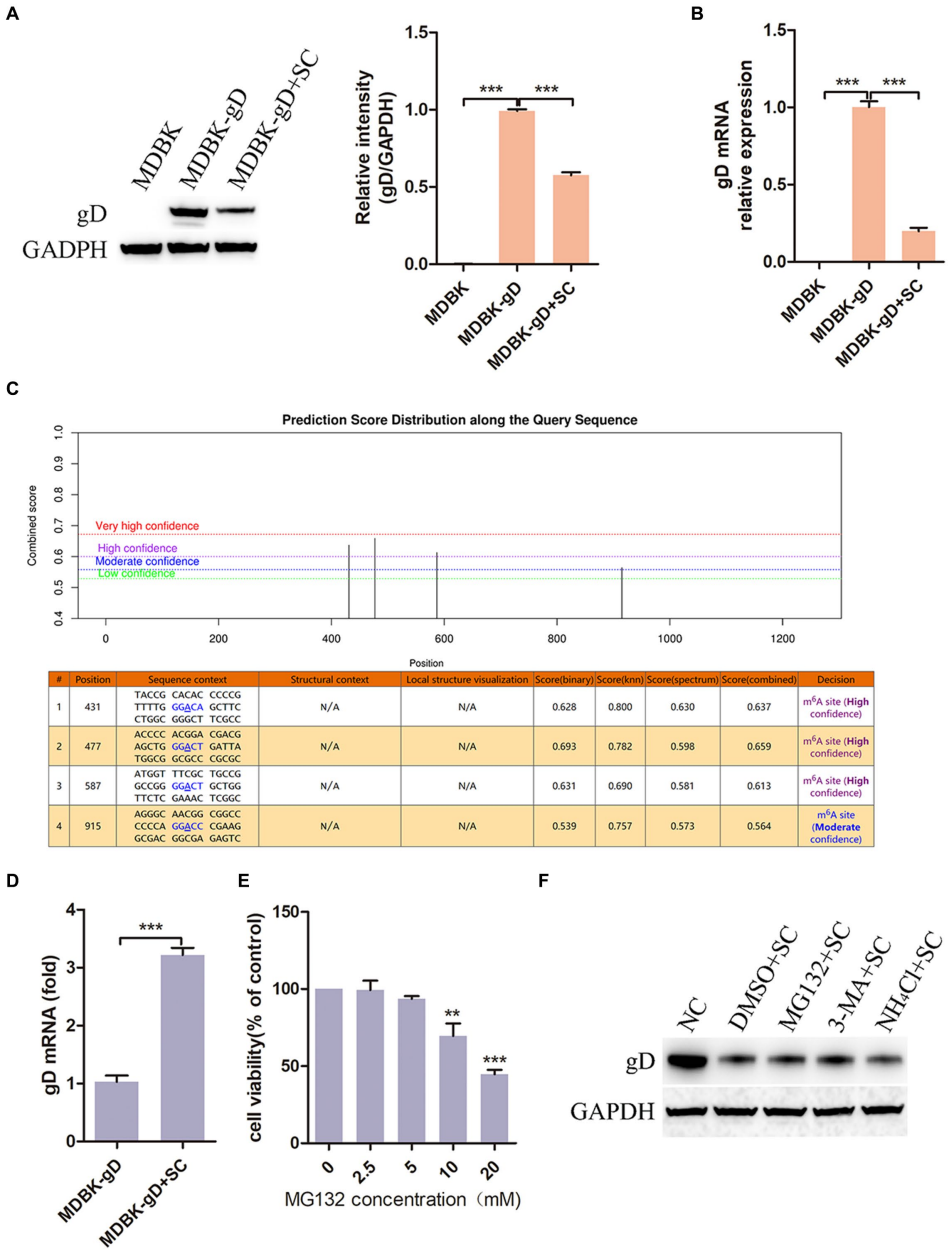
Schisandra chinensis inhibits BoHV-1 gD transcription. **(A)** MDBK-gD cells were treated with SC for 24 h at 37°C, gD expression was examed by western blot and the relative gray level was analyzed, **(B)** gD expression was analyzed by qPCR. **(C)** m6A modification sites in the coding region of gD was predicted by SRAMP. **(D)** MDBK-gD cells were treated with SC for 1 h at 37°C, followed by bound with BoHV-1 at an MOI of 2 for 24 h at 37°C in the presence of SC, m6A methylation of gD was analyzed by MeRIP-qPCR. **(E)** Viability of MDBK cells treated with MG132 were analyzed by CCK-8 reagent. **(F)** MDBK-gD cells were treated with DMSO/MG132/3-MA/NH_4_Cl and SC for 24 h at 37°C, gD expression was analyzed by western blot. **p < 0.01 and ***p < 0.001.

In the above investigation results, we observed that Schisandra chinensis had an inhibitory effect on BoHV-1 replication, prompting further investigation into its impact on viral proteins expression. We constructed a cell, MDBK-gD, expressing BoHV-1 gD protein by lentivirus packaging. Subsequent treatment of MDBK-gD cells with Schisandra chinensis to detect the effect of Schisandra chinensis on gD expression. The results of western blot and gray scale analysis showed that gD protein could be expressed in MDBK-gD cells, indicating that the cell was successfully constructed. After adding Schisandra chinensis to MDBK-gD cells, the protein expression of gD was significantly inhibited (Figure 7A). Protein expression can be influenced by transcriptional or post-translational modifications (Skelly et al., 2016; Gao et al., 2023). To investigate the impact of Schisandra chinensis on gD protein degradation, we examined gD mRNA levels. qPCR result indicated a significant decrease in gD transcription following Schisandra chinensis treatment (Figure 7B), suggesting that Schisandra chinensis may degrade gD at the transcriptional level. N6-methyladenine (m6A) methylation is a common modification in eukaryotic mRNAs, affecting mRNA stability, translation, and export (Wang et al., 2014). Using SRAMP, we analyzed four m6A modification sites (431, 477, 587, and 915 bp) in the coding region of gD (Figure 7C), and assessed the impact of Schisandra chinensis on m6A methylation of gD. MeRIP-qPCR results confirmed that Schisandra chinensis enhanced the m6A methylation level of gD (Figure 7D). Given the known prime post-translational protein degradation pathways of in eukaryotic cells are the ubiquitin-proteasome system and the autolysosome pathway (Zhai et al., 2023), we further explored whether Schisandra chinensis influenced the degradation of gD protein via these two pathways utilizing proteasome inhibitor MG132, autophagy inhibitor 3-MA, and lysosome inhibitor NH_4_Cl. The optimal working concentration of MG132 was detected, the results of cell viability assay showed that MG132 higher than 5 mM could inhibit the viability of MDBK cells (Figure 7E). We added these three inhibitors and Schisandra chinensis in MDBK-gD cells, respectively, to measure the ectopic expression of gD protein. The results of western blot analysis indicated that Schisandra chinensis did not impact the expression of gD protein (Figure 7F). These findings collectively indicate that Schisandra chinensis primarily inhibits BoHV-1 gD transcription through enhancing m6A methylation.

### 3.8 Schisandra chinensis inhibits the entry of progeny virus into cells

**Figure 8:**
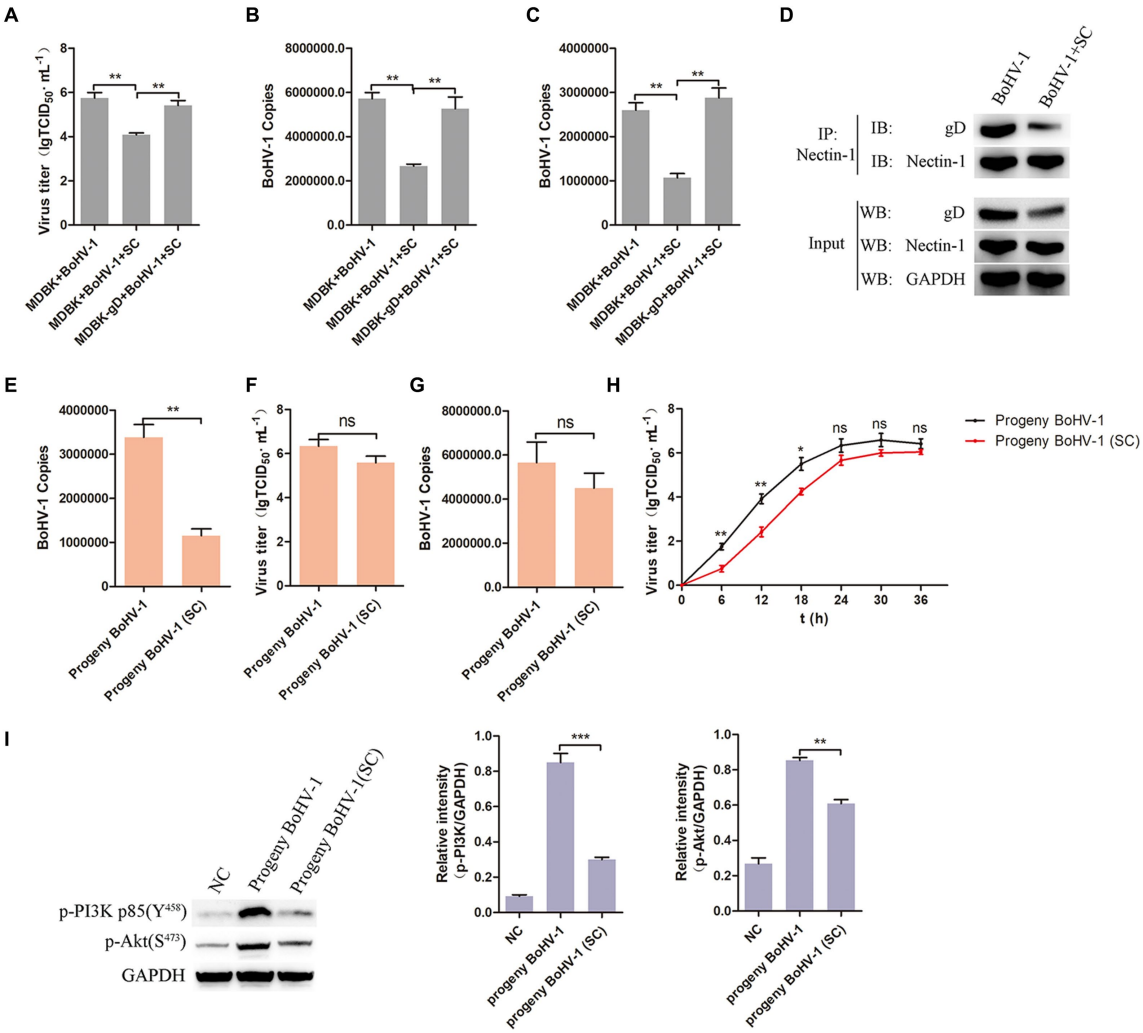
Schisandra chinensis inhibits the entry of progeny virus into cells. **(A)** MDBK and MDBK-gD cells were treated with SC for 1 h at 37°C, followed by bound with BoHV-1 at an MOI of 2 for 1 h at 4°C. Subsequently, the cells were transferred to 37°C for 24 h in the presence of SC, and virus replication was assessed by virus titer. **(B)** Virus replication was assessed by virus copies. **(C)** MDBK and MDBK-gD cells were treated with SC for 1 h at 37°C, followed by bound with BoHV-1 at an MOI of 10 for 1 h at 4°C. Subsequently, the cells were transferred to 37°C for 1 h in the presence of SC, and virus entry was assessed by virus copies. **(D)** MDBK cells were treated with SC for 1 h at 37°C, followed by bound with BoHV-1 at an MOI of 2 for 24 h. The cells were lysed and immunoprecipitated with the anti-nectin-1 antibody, and the total cell lysates were analyzed by antibody of anti-gD, anti-nectin-1 and anti-GAPDH. **(E)** MDBK cells treated with SC for 1 h at 37°C were infected with BoHV-1 at an MOI of 2 for 24 h in the presence of SC, infected MDBK cells with BoHV-1 in the absence of SC as the control group, collected progeny virus from experimental and control groups. MDBK cells were infected with the progeny viruses at an MOI of 10, the entry of the progeny virus was detected by virus copies; **(F)** MDBK cells were infected with the progeny viruses at an MOI of 2, the replication of the progeny virus was detected by virus titer, **(G)** and the replication of the progeny viruses were detected by virus copies. **(H)** MDBK cells were incubated with the progeny viruses at an MOI of 2 at 37°C, supernatants were collected for virus titer analysis at the indicated times. **(I)** MDBK cells were incubated with the progeny viruses from experimental and control groups at an MOI of 10 at 37°C for 30 min, collected the cells to analyze proteins expression, and analyze the relative gray level. **p < 0.01 and ***p < 0.001.

To further investigate the impact of Schisandra chinensis inhibiting gD transcription in BoHV-1 replication, MDBK and MDBK-gD cells were treated with Schisandra chinensis followed by BoHV-1 inoculation, and virus replication was assessed. Results from viral titer and viral copies analyses indicated that the presence of gD in MDBK cells counteracted the inhibitory effects of Schisandra chinensis on viral replication (Figures 8A,B). Moreover, our experiments demonstrated that ectopic expression of gD protein also mitigated the inhibitory effects of Schisandra chinensis on BoHV-1 entry (Figure 8C). During BoHV-1 entry into MDBK cells, gD binds to the cell receptor nectin-1 and activates the PI3K-Akt signaling pathway, leading to the expression of CHC and dynamin-2 to facilitate virus entry. We hypothesized that the presence of Schisandra chinensis leads to the reduction in gD protein expression, thereby decreasing the gD which interacted with nectin-1. This reduction subsequently hinders the activation of the PI3K-Akt signaling pathway, ultimately inhibiting progeny virus entry and virus replication. To test this hypothesis, we examined the interaction between gD protein and nectin-1 both before and after treatment with Schisandra chinensis. Indeed, the result of co-immunoprecipitation experiments demonstrated the decrease in gD protein expression following Schisandra chinensis treatment, leading to the decrease of the gD which interacted with nectin-1 (Figure 8D). In addition, we infected MDBK cells with BoHV-1 in the presence of Schisandra chinensis, and infected MDBK cells with BoHV-1 in the absence of Schisandra chinensis as a control group, collected progeny virus from experimental and control groups, infected MDBK cells with the progeny virus, and detected progeny virus entry and replication. The detection of virus copies proved that the entry of progeny BoHV-1 treated with Schisandra chinensis into cells was significantly inhibited (Figure 8E). However, Schisandra chinensis treatment did not impact progeny BoHV-1 replication (Figures 8F,G). We also examined the growth curve of progeny virus from experimental and control groups, growth curve analysis revealed that Schisandra chinensis treatment hindered early infection of progeny virus but had minimal effect on replication post-entry (Figure 8H). Further investigation included inoculation of MDBK cells with the above two progeny virus, with non-infected cells as controls. PI3K-Akt signaling pathway activation during early stage of virus infection was analysed, the result indicated that the expression of p-PI3K and p-Akt were attenuated after infecting with the progeny virus which treated with Schisandra chinensis (Figure 8I). Overall, Schisandra chinensis inhibits the entry of progeny virus into cells.

## 4 Discussion

Schisandrae chinensis, a traditional Chinese medicine with a history of over 2000 years, has been officially recognized in various editions of the Chinese pharmacopoeia and is commonly utilized in traditional Chinese medicine formulations (Li Z. et al., 2018; Szopa et al., 2018; Peng et al., 2022). Known for its antitussive and sedative properties, Schisandrae chinensis has demonstrated significant antitussive effects *in vivo* (Zhong et al., 2016, 2021). Studies have also indicated that Schisandra polysaccharide (SPJ) exhibits inhibitory effects on influenza A virus both *in vitro* and *in vivo* (Qi et al., 2023), schinlignan G and methylgomisin O have shown potential in combating hepatitis B virus (Xue et al., 2015). BoHV-1 is an enveloped DNA virus belonging to a member of the α-herpesvirus subfamily. BoHV-1 infection in cattle can cause upper respiratory tract disease, manifested as cough and dyspnea, causing significant economic losses to the global cattle industry (Turin et al., 1999; Jones and Chowdhury, 2007). Currently, the impact of Schisandra chinensis on BoHV-1 infection remains unclear. This study aims to investigate the effects and mechanisms of Schisandra chinensis on BoHV-1 infection of MDBK cells *in vitro*.

The study investigated the impact of Schisandra chinensis on BoHV-1 replication in MDBK cells. It was found that Schisandra chinensis effectively inhibited BoHV-1 replication, regardless of the infection dose of virus. In virus infection process, virus initially attaches to the cell surface, binds to the cell receptor, and then enters the cell for replication and proliferation (Keil et al., 1984; Wang et al., 2016). Our study explored the influence of Schisandra chinensis on BoHV-1 attachment and entry. Results demonstrated that while Schisandra chinensis did not affect the attachment of BoHV-1, it hindered the entry of the virus into MDBK cells.

The most common manner of virus entry into cells is endocytosis (Mercer et al., 2010), lots of enveloped viruses enter host cells through this way (Más and Melero, 2013; Nicola, 2016), such as Pseudorabies virus (PRV), Canid herpesvirus 1 (CHV-1) and BoHV-1 (Lv et al., 2018; Eisa et al., 2021; Liu et al., 2023). The acidic environment of endosomes is necessary for endocytosis (Más and Melero, 2013; Nicola, 2016). We therefore investigated the role of endocytosis in the inhibition of BoHV-1 entry and replication by Schisandra chinensis using two inhibitors of endosomal acidification, CQ and NH_4_Cl. The results proved that Schisandra chinensis block BoHV-1 entry and replication by inhibiting endocytosis. Autophagy and apoptosis are common processes of programmed cell death, playing a crucial role in disease occurrence and development (Li M. et al., 2018; Yeganeh et al., 2018; Deng et al., 2022). Subsequently, the effects of apoptosis and autophagy on Schisandra chinensis inhibiting BoHV-1 replication was also investigated. The results indicated that apoptosis and autophagy did not contribute to Schisandra chinensis inhibiting BoHV-1 replication.

Various lignans, such as Meth O, Ange H, Rubr A, Schi B, and Wuwe E, can be extracted from Schisandra chinensis (Xue et al., 2015). In this study, we investigated the inhibitory effects of these lignans on BoHV-1 entry into MDBK cells. Our findings revealed that only Meth O was able to inhibit BoHV-1 entry. Furthermore, our experiments using endosomal acidification inhibitors, CQ and NH_4_Cl, demonstrated that Meth O blocks BoHV-1 entry by inhibiting endocytosis. Previous studies have indicated that Meth O displays strong anti-hepatitis B virus (HBV) activity by impeding HBV DNA replication (Xue et al., 2015), suggesting that Meth O may serve as a promising compound with broad antiviral properties against various viral infections through distinct mechanisms.

The endocytosis can be categorized into clathrin-mediated endocytosis, caveolin-mediated endocytosis and macropinocytosis based on the proteins involved (Mercer et al., 2010). Clathrin-heavy chain (CHC) and caveolin-1 are the primary functional proteins in clathrin-mediated and caveolin-mediated endocytosis, respectively. Dynamin is an important regulator of these two pathways, which separate the invaginated vesicle from the cell membrane and helping virus to efficiently transport into cytoplasm (Orth et al., 2002; Sun and Tien, 2013). Our findings demonstrated that Meth O suppressed the increase of p-caveolin-1, CHC and dynamin-2 induced by BoHV-1 infection, consequently inhibiting clathrin-mediated and caveolin-mediated endocytosis (Liu et al., 2023). During α-herpesvirus entry, the presence of gC protein enables viral attachment to the cell surface; in the absence of gC protein, gB protein perform this function compensatively (Campadelli-Fiume et al., 2000, 2007; Li M. et al., 2018). Subsequently, gD protein binds to nectin-1, activating PI3K-Akt and p38 MAPK pathways, leading to increased expression of p-caveolin-1, CHC and dynamin-2, and activation of clathrin-mediated and caveolin-mediated endocytosis, facilitating BoHV-1 entry into host cells through both pathways (Mettenleiter, 2002; Spear, 2004; Li M. et al., 2018; Liu et al., 2023). We investigated the impact of Schisandra chinensis on PI3K-Akt and p38 MAPK pathways in the early stage of BoHV-1 infection. Indeed, our results revealed that Schisandra chinensis could block the activation of the PI3K-Akt pathway triggered by BoHV-1 infection, consequently inhibiting clathrin-mediated and caveolin-mediated endocytosis, ultimately hindering BoHV-1 entry.

Our findings indicate that Meth O functions as an inhibitor of the endocytosis, effectively inhibiting both clathrin-mediated and caveolin-mediated endocytosis. Notably, its mechanism differs from established endocytosis inhibitors. For instance, CQ and NH_4_Cl disrupt endocytosis by impeding endosomal acidification (Adachi et al., 2007; Al-Bari, 2017; Ponsford et al., 2021; Shang et al., 2021), MβCD and nystatin hinder caveolin-mediated endocytosis by depleting cholesterol from caveolae on cell membranes (Vela et al., 2007; Zhang et al., 2018; Gao et al., 2019; Pan et al., 2021), but Meth O inhibits clathrin-mediated and caveolin-mediated endocytosis by suppressing the activation of the PI3K-Akt pathway and downregulating the expression of p-caveolin-1, CHC and dynamin-2. Furthermore, considering the pivotal role of the PI3K-Akt signaling pathway in Multiple viral infections (Dunn and Connor, 2012; Bossler et al., 2019; Lahon et al., 2021; Fattahi et al., 2022; Zhang et al., 2022), Meth O may hold promise as a broad-spectrum antiviral drug, warranting further investigation into its efficacy against other viruses.

NF-kB serves as a downstream transcription factor of the PI3K-Akt signaling pathway, influencing the nuclear translocation of NF-kB and activation of clathrin-mediated and caveolin-mediated endocytosis during BoHV-1 entry into MDBK cells. Mislocalized or unmasked host RNA acts as a potent RLR agonist in DNA virus-infected cells, triggering NF-kB activation and subsequent IFN-I secretion. While it was initially hypothesized that Schisandra chinensis could inhibit IFN-I secretion by impeding NF-kB nuclear translocation, experimental results showed no impact on IFN-α and IFN-β production induced by BoHV-1 infection. This result may be due to the activation of not only RIG-I/MDA5-MAVS-TBK1-NF-kB signaling pathway, but also RIG-I/MDA5-MAVS-TBK1-IRF3 signaling pathway by cellular RNA during DNA virus infection. Upon recognition of BoHV-1 DNA, cells can also activate cGAS-STING-TBK1-IRF3 signaling pathway to promote IFN-I secretion (Fitzgerald et al., 2003; Sharma et al., 2003; Sun et al., 2013), so that the overall IFN-I secretion was not affected. Subsequent investigation of protein expression in the RIG-I/MDA5-MAVS-TBK1-IRF3 and cGAS-STING-TBK1-IRF3 pathways in Schisandra chinensis confirmed this hypothesis.

The findings from previous experiments indicate that Schisandra chinensis has the ability to inhibit BoHV-1 replication. Our hypothesis suggested that BoHV-1 might hinder viral replication by influencing the synthesis of viral proteins. Our results confirmed that Schisandra chinensis primarily impeded gD protein synthesis by suppressing gD gene transcription. Detailed mechanism revealed that Schisandra chinensis blocked BoHV-1 gD transcription through enhancing m6A methylation of gD.

Subsequent findings revealed that the overexpression of gD protein blocked the inhibitory impact of Schisandra chinensis on BoHV-1 replication and entry. It’s probably because that during BoHV-1 entry, ectopic expression of gD protein specifically interacts with the cellular receptor nectin-1 and triggers the PI3K-Akt and p38 MAPK signaling pathways (Liu et al., 2023), which counteracts the suppressive influence of Schisandra chinensis on the PI3K-Akt signaling pathway and BoHV-1 entry. Furthermore, we hypothesized that adding Schisandra chinensis leads to the decrease of gD which interacted with nectin-1, resulting in diminished activation of PI3K-Akt signaling pathways, which inhibits clathrin-mediated and caveolin-mediated endocytosis, thereby impeding progeny virus entry into cells to block replication. Experimental validation showed that Schisandra chinensis treatment indeed weakened progeny virus entry into cells without significantly affecting replication, and also attenuated the activation of PI3K-Akt pathway. These findings collectively support the conclusion that Schisandra chinensis hinders virus replication by targeting gD transcription.

## 5 Conclusion

In conclusion, Schisandra chinensis extract Meth O has been found to inhibit the entry of BoHV-1 into MDBK cells by blocking the activation of the PI3K-Akt signaling pathway to weakening clathrin-mediated and caveolin-mediated endocytosis. Following BoHV-1 entry, Schandschandra chinensis is able to suppress BoHV-1 gD gene transcription, impede BoHV-1 gD protein synthesis and progeny virus into MDBK cells to hinder virus replication.

## Data availability statement

The original contributions presented in the study are included in the article/supplementary material, further inquiries can be directed to the corresponding author.

## Author contributions

YL: Investigation, Methodology, Validation, Visualization, Writing – original draft. KW: Investigation, Methodology, Validation, Visualization, Writing – original draft. XG: Supervision, Validation, Visualization, Writing – review & editing. WQ: Validation, Visualization, Writing – review & editing. YX: Validation, Visualization, Writing – review & editing. HS: Validation, Visualization, Writing – review & editing. JK: Validation, Visualization, Writing – review & editing. JS: Validation, Visualization, Writing – review & editing. FW: Methodology, Supervision, Validation, Visualization, Writing – review & editing. FD: Methodology, Resources, Supervision, Validation, Visualization, Writing – review & editing.
